# Are You Able to Trust Me? Analysis of the Relationships Between Personality Traits and the Assessment of Attractiveness and Trust

**DOI:** 10.3389/fnhum.2021.685530

**Published:** 2021-07-26

**Authors:** Bernadetta Bartosik, Grzegorz M. Wojcik, Aneta Brzezicka, Andrzej Kawiak

**Affiliations:** ^1^Chair of Neuroinformatics and Biomedical Engineering, Institute of Computer Science, Maria Curie-Sklodowska University, Lublin, Poland; ^2^Neurocognitive Research Center, SWPS University of Social Sciences and Humanities, Warsaw, Poland; ^3^Polish-Japanese Academy of Information Technology, Warsaw, Poland

**Keywords:** trust and distrust, trust and reputation management, credibility, regress algorithm, machine learning

## Abstract

Behavioral and neuroimaging studies show that people trust and collaborate with others based on a quick assessment of the facial appearance. Based on the morphological characteristics of the face, i.e., features, shape, or color, it is possible to determine health, attractiveness, trust, and some personality traits. The study attempts to indicate the features influencing the perception of attractiveness and trust. In order to select individual factors, a model of backward stepwise logistic regression was used, analyzing the results of the psychological tests and the attractiveness and trust survey. Statistical analysis made it possible to select the most important personality traits related to attractiveness and trust assessments.

## 1. Introduction

The face is like a book that allows you to obtain information that is an important element of social communication. Watching the faces of strangers, people make social (attractiveness, credibility, intelligence, dominance) and personality (gender, age, emotions) assessments based on facial readings (Kościński, 2007; Oosterhof and Todorov, 2008, 2009; Zebrowitz and Montepare, 2008). The information obtained is particularly important in the process of analyzing the degree of credibility (Wierzbicki, 2008). It has been noted that attractive people are more often seen as trustworthy (Shinners, 2009). In addition, credibility assessment is coupled with expressed emotions. Happy and smiling faces are more credible in contrast to sad or angry faces (Sutherland et al., 2017). Scientists have shown that women and people with children's facial features have higher trust (Buchan et al., 2008; Zebrowitz et al., 2015).

It is believed that the ability to recognize faces develops automatically from an early age and is improved with development (De Heering et al., 2012; Jessen and Grossmann, 2019; Mondloch et al., 2019). Depending on the circumstances, the human brain can detect faces in just over 100 ms (Crouzet et al., 2010; Martin et al., 2018). This is important in the situations requiring an immediate decision. It is worth adding that the first impressions regarding the seen face may appear about 33 ms after the stimulus exposure (Bar et al., 2006; Freeman et al., 2014). Recently, scientists have presented views on the stage processing of the face. Visual features related to facial recognition (among others, gender and age) are recognized the fastest, followed by identity identification (di Oleggio Castello and Gobbini, 2015; Dobs et al., 2019).

Facial credibility has been proven to be essential to the trust that influences cooperation (Zebrowitz and Montepare, 2008). Murderers whose faces are trustworthy are more likely to get milder punishments (Wilson and Rule, 2015; Ancāns and Austers, 2018). Based on the assessment of the credibility of the face, scientists are able to predict the results of political choices (Ballew and Todorov, 2007). Face perception also has a big impact on online sales, when buyers are more likely to choose the offer of a seller with a more reliable face, regardless of the issued reviews (Ert et al., 2016). Similarly in the “trust games,” people are more likely to spend money on a trustworthy partner, and the amount of the stake depends on the level of trust (Van't Wout and Sanfey, 2008; Chang et al., 2010).

Perception of socially important stimuli relies on the temporal cortical areas of the temporal lobe, and their connection with emotions and motivation is provided, among others, by the amygdala, orbitofrontal cortex, or cingulate cortex (Adolphs, 2001). Numerous brain tests have shown that the amygdala is one of the most important regions during the credibility assessment process (Engell et al., 2007; Todorov et al., 2008). The amygdala activity is associated with the processing of lower level emotional stimuli. It increases its activity during social assessment based on the emotional state and intentions of others (Costafreda et al., 2008). Depending on the degree of credibility of the face, the amygdala is more or less activated, with reliably looking faces reducing activity, and a decrease in credibility causing an increase in its activity (Haas et al., 2015).

Appearance is one of numerous characteristics that can influence the initiation or continuation of a relationship with another person. It can also affect the level of trust in the other person. As a matter of fact, there are so many canons of beauty and criteria for choosing in people. The aim of the research was to show the relationship between the personality traits and the assessment of trust and attractiveness toward the faces shown in the photos. For this purpose, there were carried out a survey to examine whether the faces of the people in the photos inspire trust and whether they are attractive as well as two psychological tests: the IVE Impulsivity Questionnaire and the NEO PI-R Personality Inventory. Both tests are used to diagnose personality traits. Using IVE you can define three features (impulsiveness, risk-aversion, empathy), and through NEO PI-R five (neuroticism, extrovertness, openness to experience, agreeableness, conscientiousness), each of these features has six more elements. After applying the stepwise backward logistic regression model, only those features that had the most significant impact on the dependent variables, i.e., attractiveness and trust, were selected. To our knowledge, this is one of the first EEG protocols planned on this subject. The current study is a pilot for further research using EEG.

## 2. Tools

Personality traits were examined using the NEO PI-R personality questionnaire by the authors of McCrae and Costa, in the Polish translation of Siuta. The NEO PI-R questionnaire is a test modeled on the five-factor personality model (Big Five), which takes into account five main dimensions (Costa and McCrae, 1992):

- neuroticism—a dimension defined by fear, guilt, dissatisfaction, anger (high neuroticism). Susceptibility to negative feelings causes weaker control over emotions, increases stress, and leads to illogical behavior. Low neuroticism characterizes emotionally stable, calm, and composed people.
- extroversion—it defines people prone to social interactions, able to feel positive emotions, active, and energetic. Extroverts are friendly to others, talkative, focused on searching for new stimuli. The opposite and at the same time the opposite end of the scale of this dimension is introversion, which characterizes less daring, more secretive people and avoiding such active social contacts.
- openness to experience—expresses a tendency to look for new life experiences. People with high openness are curious about the world, more tolerant and easily take on new tasks.
- agreeableness—defined as an attitude toward other people. A high level of agreeableness is characterized by trust in others, honesty, and a disinterested willingness to help. Agreeableness in a pejorative version reflects egocentrism, aggression, and dry relationships with other people.
- conscientiousness—described by such values as conscientiousness, punctuality, and diligence. High conscientiousness is primarily duty, goal-oriented action, high motivation, but also perfectionism or excessive dedication to work. On the other side of the conscientiousness scale, there are no defined life goals, low motivation to act and spontaneity as well as impulse decision-making.

The first personality inventory consisted of three factors, each of which had six subscales. On the basis of numerous observations, the model was extended with two further features and only modified in the following years. The current test consists of the following trait factors: neuroticism, extroversion, openness, agreeableness, and conscientiousness. Each of them is divided into six subscales. The worksheet consists of 240 questions. The respondent's task is to answer questions on a scale of 0–4 depending on how true the question is for the participant.

The Impulsiveness Questionnaire (IVE) by Hans J. Eysenck and Sybil B. G. Eysenck is a test used to diagnose personality traits in adults and high school students. It consists of three scales:

- the scale of impulsivity characteristic of people who make decisions without thinking about their effect,
- the scale of empathy, describing people sensitive to other people's emotions along with an adequate action,
- the scale of propensity to risk typical of people willing to take on new challenges.

The sheet contains 54 questions to which the respondent answers yes by marking “YES” or marking “NO” in the negative. The result for individual scales is the sum of points scored for the answers belonging to them (Caci et al., 2003).

From the available databases, two relatively large and generally available resources were selected, which are intended for the development of science. Both sets are characterized by good resolution and accuracy of the taken photos. Both databases are designed to provide users with standardized and multi-aspect-tested photo sets that have been used in other facial processing studies (Silver et al., 2020; Assem et al., 2021). The first is the Development Emotional Faces Stimulus Set (DEFSS) which contains the total of 404 face pictures showing different emotions such as sadness, happiness, fear, anger, and neutral facial expressions. The models were people aged 8–30, mostly white. An additional advantage is the verification of photos made by the creators, during which independent respondents and photographed people assessed the presented emotions (Meuwissen et al., 2017). The second is the Multi-Racial Mega-Resolution (MR2) which shows the photos of 74 people between the ages of 18 and 25. Contrary to the previous database, it presents the photos of various races people (European, African, and East Asian), with only a neutral facial expression without makeup (Strohminger et al., 2016).

**Figure 1:**
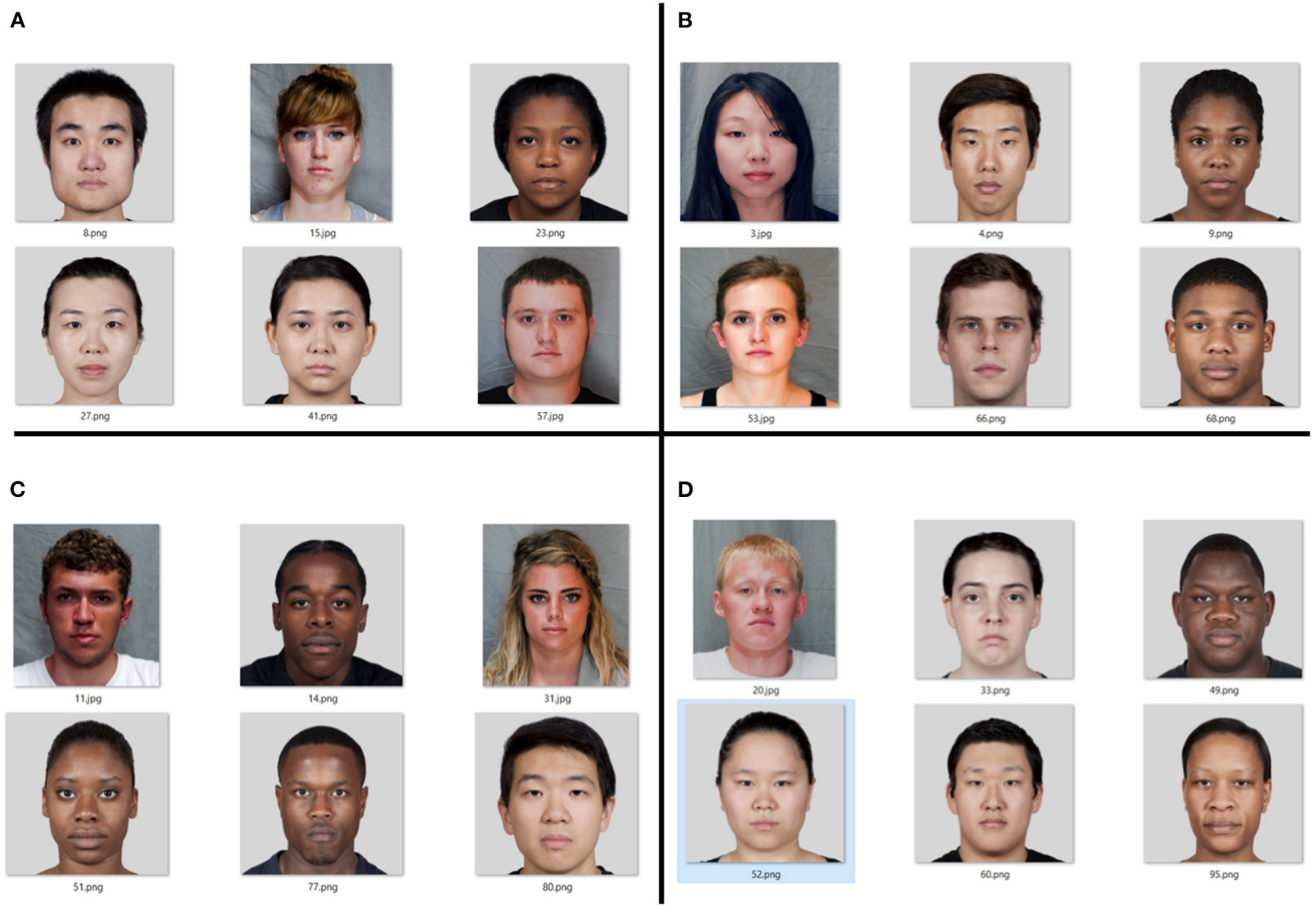
A set of photos selected for future research, divided into groups: **(A)** low attractiveness and high confidence, **(B)** high attractiveness and high confidence, **(C)** high attractiveness and low confidence, **(D)** low attractiveness and low trust. Photos taken from the following databases: DEFSS (Meuwissen et al., 2017) and MR2 (Strohminger et al., 2016).

One hundred photos (50 women and 50 men) were selected from the two sets. Only photographs showing the face from the front without emotions (with a neutral expression on the face) were taken into account during the selection. Overall, 49 photos show the people of European descent, 31 photos the people of African descent, and 20 photos those of East Asian descent. A questionnaire was made for the obtained base, in which three questions were displayed for each photo. The first question concerned the gender of the person in the photo and the respondent chose a woman and a man from the answers. The second and third questions were about attractiveness and trust, respectively. Using a five-point scale, the participants determined to what extent the person in the photo is attractive and to what extent they can trust the person in the photo, where in both questions 1 meant not at all, and 5 very much. Eighty-five students of computer science and cognitive science at Maria Curie-Skłodowska University were invited to participate in the survey, and their answers were statistically analyzed. The photos are divided into four groups: attractive and trustworthy, unattractive and untrustworthy, attractive and untrustworthy, unattractive and trustworthy. Within these groups, based on the division by sex and origin of the people in the photos, six photos were selected for each group (Figure 1), rated the highest by the respondents. As a result, 24 photos were selected for future study.

## 3. Procedure

As part of additional activity in the classroom, 85 students of Maria Curie-Skłodowska University enrolled in the research. The participants were mainly first-year students of cognitive science and first-year computer science, aged 18–24. Taking care of the confidentiality of personal data and the comfort of the participants, the laboratory employees generated random logins and passwords, which were used by the students to identify themselves while completing the questionnaires ensuring their anonymity.

The study consists of two psychological tests and a face survey. The first test in each case was the NEO PI-R. The test consisted of 240 questions, the participant assigned an answer to each question from 0 to 4 depending on how much he agreed with the statement. The next test was IVE consisting of 54 questions with the possibility of answering “yes” or “no.” The last one was a questionnaire containing 100 photos of faces. For each photo, three questions were displayed in turn: “What is the gender of the person in the photo,” “How attractive is the person in the photo,” “To what extent are you able to trust the person in the photo.” The participant chose the answer to the first question, a woman or a man, and chose the next two on a scale from 1 to 5 where 1 meant not at all and 5 very much.

## 4. Description of the Statistical Method

The statistical analysis was performed using the logistic regression model in the SPSS program. It is a type of nonlinear analysis that allows you to describe the direction and strength of the relationship between individual explanatory variables in quantitative or qualitative form and dichotomous dependent variables that assume values of 0 or 1. The model was composed of 34 explanatory variables which include of personality traits tested with the NEO PI-R and IVE psychological tests. Due to the characteristics of logistic regression and the desire to extract the variables as well as possible,the dependent variables of the model are attractiveness and trust represented by a dichotomous variable where 1 means that the respondent assessed the face from the photo as attractive/trustworthy, 0 means that the respondent assessed the face in the photo negatively. The data set introduced into the model did not include the division into training and test sets. In addition, it is worth mentioning that the presented study is a pilot for further research in which ERP will be tested using EEG in the context of trust/not trust.

The designed backward stepwise logistic regression model took into account additionally the Wald criterion to optimize the number of variables influencing the dependent variables. The higher Wald's coefficient, the more influencing on attractiveness and trust the variable is. A stepwise regression model was used, which means that the model gradually changed. In this case, along with the next step, one variable with the lowest value of Wald's criterion was rejected from the model and further statistical activities were carried out on the remaining data set (Table 1). The number of explanatory variables decreased to 5 in the attractiveness model which obtained satisfactory results in 30 steps (Nagellerke's R2 = 0.261) and to 9 in the confidence model with the number of steps equal to 26 (Nagellerke's R2 = 0.434).

**Table 1:** Summary of the most important explanatory variables (marked with a “+” in the table) for the variables attractiveness and trust, which remained after elimination by backward logistic stepwise regression based on the Wald coefficient.

**Variable**	**Attractiveness**	**Trust**
N1- Anxiety	−	−
N2- Angry hostility	+	−
N3- Depression	−	−
N4- Self-consciousness	+	−
N5- Impulsiveness	+	−
N6- Vulnerability	−	−
E1- Warmth	−	−
E2- Gregariousness	−	−
E3- Assertiveness	−	−
E4- Activity	+	−
E5- Excitement seeking	−	−
E6- Positive emotions	−	−
O1- Fantasy	−	−
O2- Aesthetics	−	−
O3- Feelings	−	−
O4- Actions	+	−
O5- Ideas	−	+
O6- Values	−	−
U1- Trust	+	−
U2- Straightforwardness	−	−
U3- Altruism	+	+
U4- Compliance	−	−
U5- Modesty	−	−
U6- Tendermindedness	−	−
S1- Competence	−	−
S2- Order	−	+
S3- Dutifulness	−	−
S4- Achievement striving	−	−
S5- Self-discipline	−	−
S6- Deliberation	−	−
Empathy	+	+
sdr-Tendency to take risks	−	+
Impulsiveness	+	−
constant	+	+

## 5. Correctness of the Logistic Model

Principal Component Analysis (PCA) is one of the statistical methods that examines data without supervision. The main goal is to reduce the number of input variables describing the phenomenon under study. By design, this method is used to explain the variability of complex data using new principal components that are linear combinations of the observed variables. The new set is characterized basing on most of the data from the original set with a reduced number of variables. The PCA analysis can be an introduction before proceeding to further statistical methods, such as cluster or discrimination analysis.

In the study, the backward stepwise logistic regression eliminated, based on the Wald coefficient, the least significant explanatory variables for the model. According to the purpose of PCA, calculations were performed and the number of variables necessary to describe the phenomenon was determined and compared with the number of variables determined in the backward stepwise logistic regression model. For the calculations, a correlation matrix containing 34 explanatory variables and a constant was used. The operations were performed with the use of functions available in the programming language “R.” The PCA analysis was used to check that the logistic regression model uses a sufficient number of describing variables. Tables 2, 3 provide the PCA statistics for attractiveness and trust.

**Table 2:** Principal component analysis of a logistic regression model based on the correlation matrix for the attractiveness predictor.

	**Standard deviation**	**Proportion of variance**	**Cumulative proportion**
Comp1	4.167	0.496	0.496
Comp2	2.752	0.216	0.712
Comp3	1.858	0.099	0.811
Comp4	1.515	0.066	0.877
Comp5	1.027	0.030	0.907
Comp6	0.968	0.027	0.933
Comp7	0.874	0.022	0.955
Comp8	0.744	0.016	0.971
Comp9	0.470	0.006	0.977
Comp10	0.454	0.006	0.983

**Table 3:** Principal component analysis of a logistic regression model based on the correlation matrix for the trust predictor.

	**Standard deviation**	**Proportion of variance**	**Cumulative proportion**
Comp1	3.466	0.343	0.343
Comp2	2.783	0.222	0.565
Comp3	1.914	0.105	0.670
Comp4	1.765	0.088	0.758
Comp5	1.298	0.048	0.806
Comp6	1.171	0.039	0.845
Comp7	1.050	0.031	0.877
Comp8	0.897	0.023	0.900
Comp9	0.886	0.022	0.922
Comp10	0.740	0.016	0.938

On the basis of the Kaiser criterion, which recommends distinguishing only those factors whose eigenvalues are >1, PCA selected five components for attractiveness and seven for trust. The components for the variable “attractiveness” explain more than 90% of the variability of the input data (first component: 43.6%, second component: 21.6%, third component: 9.9%, fourth component: 6.6%, fifth component: 3%) while the components for the variable “trust” in about 87% (first component: 34%, second component: 22%, third component: 10%, fourth component: 9%, fifth component: 5%, sixth component: 4%, seventh component: 3%). The above statistical data indicate a sufficient number of components that can be used to describe the studied phenomenon. The logistic regression model, as a result of the stepwise elimination of the least statistically significant features, left five predictors for attractiveness and nine predictors for confidence. Comparing the number of predictors necessary to describe the phenomenon determined by the PCA with the number of predictors left as a result of the logistic regression model calculations, it is concluded that the model left a minimum and sufficient number of predictors to describe the attractiveness while for the description of confidence it distinguished two additional predictors above the minimum necessary.

The quality of the model was assessed using the Hosmer-Lemeshow test (Table 4). In both cases the obtained results showed no significance which proves the similarity of the observed and expected values and a good fit of the model.

**Table 4:** Goodness of fit test results from Hosmer and Lemeshow.

	**Step**	**Chi-square**	**df**	**Relevance**
Attractiveness	1	7.827	8	0.451
	30	9.075	8	0.336
Trust	1	9.612	7	0.212
	26	7.411	7	0.387

## 6. Results

As a result of the analysis of the obtained data, the impact of individual descriptive variables on dependent variables was assessed. For a better understanding of the results of the regression model, a table of correlation between the dimensions of the NEO-Pi-R questionnaire and the subscales of these dimensions is presented below. Table 5 shows only high correlations between dimensions and their subscales. The data show that the subscales do not correlate significantly with each other.

**Table 5:** Correlation between the dimensions of the NEO-Pi-R questionnaire and their subscales.

	**Neuroticism**	**Extroversion**	**Openness**	**Agreeableness**	**Conscientiousness**
N1	0.780				
E1		0.748			
S1					0.711
E2		0.877			
O2			0.742		
U2				0.739	
S2					0.711
N3	0.772				
E3		0.761			
U3				0.704	
S3					0.801
N4	0.721				
E4		0.815			
U4				0.835	
E5		0.740			
S5					0.715
N6	0.759				

The model uses a default cutoff of 0.5. The case classification derived from the logistic regression uses the predicted probability. The case with the predicted probability greater than the cutoff value is classified as positive (1), and less, as negative (0). Based on Tables 6, 7, which compare the classification of the tested values with respect to the dependent variable with the classification resulting from the use of the model, it is concluded that the model in which the dependent variable is “attractiveness” classifies correctly 72.6% of the data. In the model where the dependent variable is “trust,” the total of 78.8% of correctly predicted responses was recorded.

**Table 6:** Percentage of correct classifications in the training data for the dependent variable “attractiveness”.

**Observed**			**Predicted**		
			**Attractiveness**		**Percentage of correct classifications**
			**0**	**1**	
Step 1	Attractiveness	0	48	4	92.3
		1	5	27	84.4
	Total percentage				89.3
Step 30	Attractiveness	0	45	7	86.5
		1	16	16	50
	Total percentage				72.6

**Table 7:** Percentage of correct classifications in the training data for the dependent variable “trust.”

**Observed**			**Predicted**		
			**Trust**		**Percentage of correct classifications**
			**0**	**1**	
Step 1	Trust	0	33	7	82.5
		1	5	40	88.9
	Total percentage				85.9
Step 26	Trust	0	30	10	75
		1	8	37	82.2
	Total percentage				78.8

In order to check how the model behaves in the event of data reduction, the dimensions of the NEO-PI-R questionnaire were omitted, leaving only the subscales of dimensions in the model.

The above tables (Tables 8, 9) show that the model limitation contributed to the improvement of the model quality in the case of the dependent variable “attractiveness,” thus giving 82.1% of correct classifications. The changes in the model did not improve the classification for the dependent variable “trust.” The interpretation of the classification accuracy of 70% + may be misleading, although only the model from which the individual data is predicted was presented in the studies. The study is only a pilot and will be used for further analysis in EEG studies, the current indicators will be auxiliary indicators, while the main indicators will be indicators taken from the EEG.

**Table 8:** Percentage of correct classifications in the training data for the dependent variable “trust.”

**Observed**		**Predicted**		
		**Trust**		**Percentage of correct classifications**
		**0**	**1**	
Trust	0	30	10	75.0
	1	8	37	82.2
Total percentage				78.8

**Table 9:** Percentage of correct classifications in the training data for the dependent variable “attractiveness”.

**Observed**		**Predicted**		
		**Attractiveness**		**Percentage of correct classifications**
		**0**	**1**	
Attractiveness	0	46	6	88.5
	1	9	23	71.9
Total percentage				82.1

The values of the parameters of the regression model are presented in Table 10. As a result of the reduction in the number of variables increasing with each successive regression step, several most influential components of the model were obtained. For better illustration of the influence of individual features on the assessment of attractiveness and trust, the data are presented in Table 11. Additionally, the features influencing significantly both predictors simultaneously were marked.

**Table 10:** Estimating the values of the parameters of the logistic regression model for the dependent variable—attractiveness and trust.

		**Trust**					**Attractiveness**				
		**B**	**Standard error**	**Wald**	**Relevance**	**Exp(B)**	**B**	**Standard error**	**Wald**	**Relevance**	**Exp(B)**
Step 1	N1	−0.061	0.753	0.006	0.936	0.941	0.77	1.102	0.488	0.485	2.16
	E1	0.073	0.758	0.009	0.923	1.076	−2.149	0.967	4.941	0.026	0.117
	O1	−0.015	0.67	0	0.983	0.985	−0.11	0.828	0.018	0.894	0.896
	U1	1.347	0.811	2.759	0.097	3.848	0.719	0.748	0.925	0.336	2.053
	S1	−0.826	0.701	1.387	0.239	0.438	−2.232	1.363	2.683	0.101	0.107
	N2	2.499	0.498	6.948	0.008	12.173	0.073	0.955	0.006	0.939	1.076
	E2	−0.381	0.737	0.268	0.605	6.683	2.351	1.331	3.119	0.077	10.493
	O2	−0.566	0.754	0.564	0.453	0.568	0.261	0.918	0.081	0.776	1.298
	U2	−0.979	0.69	2.014	0.156	0.376	−1.705	1.137	2.248	0.134	0.182
	S2	0.07	0.533	0.017	0.896	1.072	−2.252	0.905	6.19	0.352	0.487
	N3	−0.246	0.603	0.166	0.684	0.782	−0.72	0.773	0.866	0.352	0.487
	E3	0.133	0.781	0.029	0.865	1.142	−1.92	1.182	2.636	0.104	0.147
	O3	−0.307	0.68	0.204	0.651	0.735	−1.339	0.936	2.048	0.152	0.262
	U3	2.266	0.817	7.687	0.006	9.643	5.185	1.753	8.746	0.003	178.51
	S3	−1.206	0.79	2.33	0.127	0.299	0.043	1.129	0.001	0.969	1.044
	N4	−1.111	0.714	2.419	0.12	0.329	−1.757	1.041	2.849	0.091	0.173
	E4	−1.168	0.755	2.391	0.122	0.311	−1.684	1.053	2.557	0.11	0.186
	O4	1.204	0.785	2.351	0.125	3.335	1.183	0.827	2.049	0.152	3.265
	U4	0.27	0.683	0.157	0.692	1.311	0.048	0.982	0.002	0.961	1.05
	S4	−0.154	0.646	0.057	0.811	0.857	2.137	1.166	3.361	0.067	8.475
	N5	−1.282	0.754	2.892	0.089	0.278	−0.188	0.835	0.051	0.821	0.828
	E5	−0.322	0.718	0.201	0.654	0.725	−2.679	1.338	4.009	0.045	0.069
	O5	−0.585	0.775	0.57	0.45	0.557	1.33	1.042	1.629	0.202	3.782
	U5	−0.72	0.661	1.187	0.276	0.487	−2.238	1.099	4.146	0.042	0.107
	S5	0.156	0.599	0.068	0.795	1.168	−1.189	0.794	2.239	0.135	0.305
	N6	−0.135	0.795	0.029	0.865	0.874	−0.358	1.094	0.107	0.744	0.699
	E6	0.417	0.669	0.389	0.533	1.517	1.075	0.891	1.456	0.227	2.93
	O6	1.176	0.726	2.622	0.105	3.24	1.496	0.822	3.307	0.069	4.462
	U6	−0.111	0.485	0.053	0.819	0.895	1.213	0.758	2.56	0.11	3.362
	S6	1.317	0.847	2.419	0.12	3.733	0.575	0.865	0.442	0.506	1.777
	impulsiveness	0.602	0.538	1.253	0.263	1.825	−1.418	0.824	2.958	0.085	0.242
	sdr	0.346	0.57	0.369	0.544	1.414	1.505	0.937	2.581	0.108	4.505
	empathy	−1.243	0.621	4.002	0.045	0.288	−1.339	0.818	2.676	0.102	0.262
	tmzS	−1.016	0.652	2.43	0.119	0.362	−1.385	0.98	1.998	0.157	0.25
	constant	0.04	0.358	0.013	0.91	1.041	−1.948	0.701	7.721	0.005	0.143
Step 26	U1	0.709	0.331	4.599	0.032	2.032	−	−	−	−	−
	N2	1.153	0.442	6.8	0.009	3.169	−	−	−	−	−
	U3	1.041	0.371	7.859	0.005	2.833	−	−	−	−	−
	N4	−0.721	0.333	4.703	0.03	0.486	−	−	−	−	−
	E4	−0.753	0.398	3.579	0.059	0.471	−	−	−	−	−
	O4	0.694	0.357	3.776	0.052	2.001	−	−	−	−	−
	N5	−0.605	0.374	2.609	0.106	0.546	−	−	−	−	−
	Impulsiveness	−0.717	0.368	3.794	0.051	2.047	−	−	−	−	−
	Empathy	−0.592	0.351	2.846	0.092	0.553	−	−	−	−	−
	Constant	0.096	0.273	0.124	0.725	1.1	−	−	−	−	−
Step 30	S2	−	−	−	−	−	−0.741	0.303	6	0.014	0.477
	U3	−	−	−	−	−	0.961	0.32	9	0.003	2.615
	O5	−	−	−	−	−	0.501	0.298	2.82	0.093	1.65
	sdr	−	−	−	−	−	0.535	0.299	3.208	0.073	1.707
	Empathy	−	−	−	−	−	−0.471	0.292	2.608	0.106	0.624
	Constant	−	−	−	−	−	−0.678	0.263	6.675	0.01	0.507

*The table shows the statistics for each of the predictors in the model. As a result of the gradual elimination of the predictors in the light parts of the table (step 26 for the confidence dependent variable, step 30 for the attractiveness dependent variable), the variables furthest away from the dependent variable are presented*.

**Table 11:** List of personality traits with the most important ones for attractiveness (marked light yellow—the positive impact on the dependent variable, dark yellow—the negative impact on the dependent variable), trust (marked light green—the positive impact on the dependent variable, dark green—the negative impact on the dependent variable) and attractiveness and trust at the same time (marked light blue—the positive impact on the dependent variables, dark blue—the negative impact on the dependent variables).

**Type of test**	**Personality factors**	**Component factors**
Neo-Pi-R	Neuroticism	N1- Anxiety
		N2- Angry hostility
		N3- Depression
		N4- Self-consciousness
		N5- Impulsiveness
		N6- Vulnerability
	Extroversion	E1- Warmth
		E2- Gregariousness
		E3- Assertiveness
		E4- Activity
		E5- Excitement seeking
		E6- Positive emotions
	Openness	O1- Fantasy
		O2- Aesthetics
		O3- Feelings
		O4- Actions
		O5- Ideas
		O6- Values
	Agreeableness	U1- Trust
		U2- Straightforwardness
		U3- Altruism
		U4- Compliance
		U5- Modesty
		U6- Tendermindedness
	Conscientiousness	S1- Competence
		S2- Order
		S3- Dutifulness
		S4- Achievement striving
		S5- Self-discipline
		S6- Deliberation
IVE	Empathy	
	sdr - Tendency to take risks	
	Impulsiveness	

Among the variables for the assessment of attractiveness, the most important is altruism (U3), which is the agreeableness subscale in the NEO PI-R test. According to the data, as the variable increases, the probability that the face in the photo will be assessed as attractive increases over 2.5 times. This variable is also the most important predictor when trust is the dependent variable in the model. In this case the probability of trust grows also more than 2.5 times with an increase in the value of the predictor. Another factor that influences the assessed attractiveness significantly is order (S2, component of conscientiousness in the NEO PI-R test). The increase in the variable enhances the chance of a positive visual assessment of the person in the photo by about 50%. In the case of empathy, which is a variable derived from the IVE questionnaire, it follows that more emphatic people are prone to negative assessment of attractiveness. The most similar results were obtained for the variables O5 (ideas, openness subscale) and sdr (propensity to risk from the IVE questionnaire). In both cases, an increase in the value of the variable leads to an increase in the chances that the respondent will evaluate the person in the photo as attractive.

As mentioned before for the dependent variable trust, a particularly strong predictor is the variable U3 or altruism. In addition to this variable, the N2 component, in other words, angry hostility from the NEO PI-R test, described as a tendency to irritation or anger, has a large impact. As follows from the data the stronger the personality trait, the greater the probability of trusting the person in the photo. Excessive self-consciousness (N4) is another variable that plays a significant role in the model. According to the presented statistics, the chances of trust by people manifesting social anxiety or low self-esteem drop by half. The group describing the dependent variable also included a feature of the same name, i.e., trust (U1) from the NEO PI-R test. When the variable changes, the probability that the face in the photo is judged trustworthy doubles. Very similar results were obtained for the variable O4 (actions from the NEO PI-R test) and impulsiveness from the IVE test. In both cases, the probability is doubled. The variable E4 (activity) has a negative component which means that its increase results in a reduction of the probability of trust by ~52%. The same relationship is found empathy from the IVE test and for the E5 variable (excitement seeking). The decrease in odds is ~44 and ~45%, respectively.

## 7. Discussion

The subject of personality and its influence on human behavior has been of interest for researchers for many years. Müller and Schwieren (2020) examined the significance of individual personality types on the behavior of participants during a trust game. They showed that personality influences human behavior based on trust with higher correlations with ambiguous decisions than with risky decisions. They indicated that neuroticists spend lower stakes during the game while people characterized by agreeableness are inclined to donate higher amounts. This statement can be translated into trust, i.e., agreeable people have a higher level of trust toward another person than in the case of neurotics. Similar conclusions were obtained by Ben-Ner and Halldorsson (2010) in the studies based also on the trust game. They found that the personality type positively influenced trust when the participant was characterized by high agreeableness, extraversion, or low neuroticism.

In the above paper, the impact of individual personality traits on the assessment of trust in people in the photos and on their attractiveness was examined. Personality traits are listed on the basis of the Neo Pi-R and IVE psychological tests and compared with the questionnaires examining the attractiveness and trust toward people in the photos. The faces obtained from generally available databases with a neutral expression were used in the research to minimize the impact of facial expression on the assessment. The analysis was performed based on the logistic stepwise backward regression. The included Wald coefficient allowed for the elimination of the least significant descriptive variables from the model with each successive step. The model was developed in the SPSS program and the learning and training of the model was carried out according to the procedures available in the program. Based on the obtained data and the performed analyses, it was shown that altruism has the greatest impact on the perceived attractiveness and trust. In both cases this trait has a positive effect on the dependent variable which indicates that altruists are more likely to judge others positively. Overall, it was noted that trust is largely influenced by the components of agreeableness and neuroticism.

After collecting the data from the respondents, apart from determining the personality traits influencing the decisions, a set of photos was extracted to be used in the electroencephalographic (EEG) examinations. The set of all 100 photos was divided into four groups: attractive and trustworthy, unattractive and untrustworthy, attractive and untrustworthy, unattractive and trustworthy. These are different combinations of dependent variables. Each of these groups has a different number of photos. The most numerous groups are great attractiveness with great confidence and small attractiveness with small confidence. The reason for this allocation of photos is the relationship between the attraction and the trust. Attractive people are believed to be more trustworthy, and less attractive people are likewise unreliable (Oosterhof and Todorov, 2008; Sutherland et al., 2017). Among the photos from the group of high attractiveness and low trust, there are only faces of people of African and European descent with a slight predominance of people of African descent. On the other hand, in the group where trust is great and attractiveness small, there are usually faces of people of Asian and European origin, with a slight predominance of the former. Based on the highest average ratings for each photo and the gender of each group, six photos were selected to be used in the EEG tests.

In the presented study, the focus was primarily on finding the relationship between the choices made toward people in the photos and personality trait. It is an introduction to further research using electroencephalography. EEG research will be developed on the above-mentioned set of faces and will be conducted on a group of 60 students.

Stimulating the brain with various types of stimuli results in the arousal coming from the centers responsible for reading and processing them. The resulting neural processes are analyzed in terms of time dynamics which are estimated using the event-related potential (ERP). Component N170 is assumed to be a component characteristic of facial perceptual processing and appears ~170 ms after the stimulus has occurred. N170 can also appear as a result of multiple face presentations and is called the adaptive effect of N170 (Eimer et al., 2010). ERP components for trust and attractiveness appear both in the initial stages of facial processing (e.g., P100) and later (e.g., late positive potential, LPP). The study (Marzi et al., 2014) analyzed, inter alia, the differences between the ERP components of trustworthy and untrustworthy faces. The stimulus responses included P100 (110–130 ms), EPN (200–350 ms), and LPP (300–500 ms). Additionally, components for trustworthy faces showed lower amplitudes than untrustworthy faces. Both early and late facial processings were recorded in the study (Yang et al., 2011) where the reliability of pre-categorized faces was analyzed and the most significant responses were obtained for the C1 (40–90 ms) and LPC (400–600 ms). Early ERP modulations occur in response to attractive/unattractive faces (Marzi and Viggiano, 2010; Hahn et al., 2016). Early registration of stimulus-induced activity is the result of facial perceptual processing. The course of facial identity processing results in the registration of the signal within the N250 limits (Werheid et al., 2007) while the signals resulting from cognitive processing that occur within 300–600 ms from the stimulus occurrence are represented the latest (Calvo et al., 2018).

Taking into account the information already available on the processing of visual stimuli by the brain in the context of attractiveness and trust, and the data obtained in the present study, an ERP analysis is planned in the next works to find the correlation between the “class” of the face and the activity of selected areas of the brain using photos of women and men different facial features and nationalities, and then, using appropriate algorithms, to accurately indicate the areas of the cerebral cortex that showed the highest activity during the experiment in individual people. As shown in the above study, the personality traits of the evaluators influence largely the decisions. In addition to the above-mentioned studies, the signal analysis is planned in terms of individual differences of respondents. The collected data in combination with EEG data will constitute a sufficient set to build a classifier that will be able to predict consensus based on personality traits.

## 8. Conclusions

On the basis of the estimated models there were demonstrated the personality traits that are most important concerning the behavior toward others, and more precisely they affect the assessment of attractiveness and trust in people from the photos significantly. Among all the features for both dependent variables, altruism (U3) is the most important. The growing probability of giving the grade “attractive” and “trustworthy” can be justified by the character traits of these people, i.e., sensitivity to the fate of another person and selfless help. It has been noticed that the components belonging to the groups of agreeableness and neuroticism have a particularly large impact on trust while altruism (U3), trust (U1), and angry hostility (N2) increase the probability of trust twice or three times and excessive self-consciousness(N4) reduces the probability by about 50%.

Future study will assess the course of ERP induced during social assessment based on the first impressions and facial appearance and locate the most active areas of the brain, detailing the Brodmann's area (BA). For this purpose an electroencephalographic test will be carried out to assess the credibility and attractiveness of the presented faces. EEG data are expected to help find a correlation between the face “class” and the activity in the selected areas of the cerebral cortex.

## Data Availability Statement

The raw data supporting the conclusions of this article will be made available by the authors, without undue reservation.

## Ethics Statement

The studies involving human participants were reviewed and approved by Maria Curie-Sklodowska University in Lublin Bioethical Commission. The patients/participants provided their written informed consent to participate in this study.

## Author Contributions

BB: project idea, manuscript writing, and surveying. GW: project idea and concept and model design. AB: psychological tests selection. AK: statistical analysis. All authors contributed to the article and approved the submitted version.

## Conflict of Interest

The authors declare that the research was conducted in the absence of any commercial or financial relationships that could be construed as a potential conflict of interest.

## Publisher's Note

All claims expressed in this article are solely those of the authors and do not necessarily represent those of their affiliated organizations, or those of the publisher, the editors and the reviewers. Any product that may be evaluated in this article, or claim that may be made by its manufacturer, is not guaranteed or endorsed by the publisher.

## References

[B1] Adolphs R. (2001). The neurobiology of social cognition. Curr. Opin. Neurobiol. 11, 231–239. 10.1016/S0959-4388(00)00202-6 11301245

[B2] Ancāns K. Austers I. (2018). The influence of face trustworthiness on judgments in forensic context. Baltic J. Psychol. 19, 100–111.

[B3] Assem M. Shashidhara S. Glasser M. F. Duncan J. (2021). Precise topology of adjacent domain-general and sensory-biased regions in the human brain. bioRxiv [Preprint]. 10.1101/2021.02.21.431622 PMC9201597 34628494

[B4] Ballew C. C. Todorov A. (2007). Predicting political elections from rapid and unreflective face judgments. Proc. Natl. Acad. Sci. U.S.A. 104, 17948–17953. 10.1073/pnas.0705435104 17959769 PMC2084277

[B5] Bar M. Neta M. Linz H. (2006). Very first impressions. Emotion 6:269. 16768559 10.1037/1528-3542.6.2.269

[B6] Ben-Ner A. Halldorsson F. (2010). Trusting and trustworthiness: what are they, how to measure them, and what affects them. J. Econ. Psychol. 31, 64–79. 10.1016/j.joep.2009.10.001

[B7] Buchan N. R. Croson R. T. Solnick S. (2008). Trust and gender: an examination of behavior and beliefs in the Investment Game. J. Econ. Behav. Organ. 68, 466–476. 10.1016/j.jebo.2007.10.006

[B8] Caci H. Nadalet L. Baylé F. J. Robert P. Boyer P. (2003). Cross-cultural study of the impulsiveness-venturesomeness-empathy questionnaire (IVE-7). Comprehens. Psychiatry 44, 381–387. 10.1016/S0010-440X(03)00105-6 14505298

[B9] Calvo M. G. Gutiérrez-García A. Beltrán D. (2018). Neural time course and brain sources of facial attractiveness vs. trustworthiness judgment. Cogn. Affect. Behav. Neurosci. 18, 1233–1247. 10.3758/s13415-018-0634-0 30187360

[B10] Chang L. J. Doll B. B. van't Wout M. Frank M. J. Sanfey A. G. (2010). Seeing is believing: trustworthiness as a dynamic belief. Cogn. Psychol. 61, 87–105. 10.1016/j.cogpsych.2010.03.001 20553763

[B11] Costa P. T. McCrae R. R. (1992). Normal personality assessment in clinical practice: the NEO personality inventory. Psychol. Assess. 4:5. 10.1037/1040-3590.4.1.5

[B12] Costafreda S. G. Brammer M. J. David A. S. Fu C. H. (2008). Predictors of amygdala activation during the processing of emotional stimuli: a meta-analysis of 385 PET and fMRI studies. Brain Res. Rev. 58, 57–70. 10.1016/j.brainresrev.2007.10.012 18076995

[B13] Crouzet S. M. Kirchner H. Thorpe S. J. (2010). Fast saccades toward faces: face detection in just 100 ms. J. Vis. 10:16. 10.1167/10.4.16 20465335

[B14] De Heering A. Rossion B. Maurer D. (2012). Developmental changes in face recognition during childhood: evidence from upright and inverted faces. Cogn. Dev. 27, 17–27. 10.1016/j.cogdev.2011.07.001

[B15] di Oleggio Castello M. V. Gobbini M. I. (2015). Familiar face detection in 180 ms. PLoS ONE 10:e0136548. 10.1371/journal.pone.0136548 26305788 PMC4549263

[B16] Dobs K. Isik L. Pantazis D. Kanwisher N. (2019). How face perception unfolds over time. Nat. Commun. 10, 1–10. 10.1038/s41467-019-09239-1 30890707 PMC6425020

[B17] Eimer M. Kiss M. Nicholas S. (2010). Response profile of the face-sensitive N170 component: a rapid adaptation study. Cereb. Cortex 20, 2442–2452. 10.1093/cercor/bhp312 20080930

[B18] Engell A. D. Haxby J. V. Todorov A. (2007). Implicit trustworthiness decisions: automatic coding of face properties in the human amygdala. J. Cogn. Neurosci. 19, 1508–1519. 10.1162/jocn.2007.19.9.1508 17714012

[B19] Ert E. Fleischer A. Magen N. (2016). Trust and reputation in the sharing economy: the role of personal photos in Airbnb. Tour. Manage. 55, 62–73. 10.1016/j.tourman.2016.01.013

[B20] Freeman J. B. Stolier R. M. Ingbretsen Z. A. Hehman E. A. (2014). Amygdala responsivity to high-level social information from unseen faces. J. Neurosci. 34, 10573–10581. 10.1523/JNEUROSCI.5063-13.2014 25100591 PMC6802589

[B21] Haas B. W. Ishak A. Anderson I. W. Filkowski M. M. (2015). The tendency to trust is reflected in human brain structure. Neuroimage 107, 175–181. 10.1016/j.neuroimage.2014.11.060 25485710

[B22] Hahn A. C. Symons L. A. Kredel T. Hanson K. Hodgson L. Schiavone L.. (2016). Early and late event-related potentials are modulated by infant and adult faces of high and low attractiveness. Soc. Neurosci. 11, 207–220. 10.1080/17470919.2015.1059361 26160142

[B23] Jessen S. Grossmann T. (2019). Neural evidence for the subliminal processing of facial trustworthiness in infancy. Neuropsychologia 126, 46–53. 10.1016/j.neuropsychologia.2017.04.025 28442339

[B24] Kościński K. (2007). Facial attractiveness: general patterns of facial preferences. Anthropol. Rev. 70, 45–79. 10.2478/v10044-008-0001-9

[B25] Martin J. G. Davis C. E. Riesenhuber M. Thorpe S. J. (2018). Zapping 500 faces in less than 100 seconds: evidence for extremely fast and sustained continuous visual search. Sci. Rep. 8, 1–12. 10.1038/s41598-018-30245-8 30127454 PMC6102288

[B26] Marzi T. Righi S. Ottonello S. Cincotta M. Viggiano M. P. (2014). Trust at first sight: evidence from ERPs. Soc. Cogn. Affect. Neurosci. 9, 63–72. 10.1093/scan/nss102 22956674 PMC3871728

[B27] Marzi T. Viggiano M. P. (2010). When memory meets beauty: insights from event-related potentials. Biol. Psychol. 84, 192–205. 10.1016/j.biopsycho.2010.01.013 20109520

[B28] Meuwissen A. S. Anderson J. E. Zelazo P. D. (2017). The creation and validation of the developmental emotional faces stimulus set. Behav. Res. Methods 49, 960–966. 10.3758/s13428-016-0756-7 27325165 PMC5173446

[B29] Mondloch C. J. Gerada A. Proietti V. Nelson N. L. (2019). The influence of subtle facial expressions on children's first impressions of trustworthiness and dominance is not adult-like. J. Exp. Child Psychol. 180, 19–38. 10.1016/j.jecp.2018.12.002 30611111

[B30] Müller J. Schwieren C. (2020). Big five personality factors in the trust game. J. Bus. Econ. 90, 37–55. 10.1007/s11573-019-00928-3

[B31] Oosterhof N. N. Todorov A. (2008). The functional basis of face evaluation. Proc. Natl. Acad. Sci. U.S.A. 105, 11087–11092. 10.1073/pnas.0805664105 18685089 PMC2516255

[B32] Oosterhof N. N. Todorov A. (2009). Shared perceptual basis of emotional expressions and trustworthiness impressions from faces. Emotion 9:128. 10.1037/a0014520 19186926

[B33] Shinners E. (2009). Effects of the “what is beautiful is good” stereotype on perceived trustworthiness. UW-L J. Undergrad. Res. 12, 1–5.

[B34] Silver B. M. Conte M. M. Victor J. D. Jones R. M. (2020). Visual search for circumscribed interests in autism is similar to that of neurotypical individuals. Front. Psychol. 11:2656. 10.3389/fpsyg.2020.582074 33192903 PMC7640760

[B35] Strohminger N. Gray K. Chituc V. Heffner J. Schein C. Heagins T. B. (2016). The MR2: A multi-racial, mega-resolution database of facial stimuli. Behav. Res. Methods 48, 1197–1204. 10.3758/s13428-015-0641-9 26311590

[B36] Sutherland C. A. Young A. W. Rhodes G. (2017). Facial first impressions from another angle: how social judgements are influenced by changeable and invariant facial properties. Br. J. Psychol. 108, 397–415. 10.1111/bjop.12206 27443971

[B37] Todorov A. Baron S. G. Oosterhof N. N. (2008). Evaluating face trustworthiness: a model based approach. Soc. Cogn. Affect. Neurosci. 3, 119–127. 10.1093/scan/nsn009 19015102 PMC2555464

[B38] Van't Wout M. Sanfey A. G. (2008). Friend or foe: the effect of implicit trustworthiness judgments in social decision-making. Cognition, 108, 796–803. 10.1016/j.cognition.2008.07.002 18721917

[B39] Werheid K. Schacht A. Sommer W. (2007). Facial attractiveness modulates early and late event-related brain potentials. Biol. Psychol. 76, 100–108. 10.1016/j.biopsycho.2007.06.008 17681418

[B40] Wierzbicki A. (2008). The case for fairness of trust management. Electron. Notes Theor. Comput. Sci. 197, 73–89. 10.1016/j.entcs.2007.12.018

[B41] Wilson J. P. Rule N. O. (2015). Facial trustworthiness predicts extreme criminal-sentencing outcomes. Psychol. Sci. 26, 1325–1331. 10.1177/0956797615590992 26162847

[B42] Yang D. Qi S. Ding C. Song Y. (2011). An ERP study on the time course of facial trustworthiness appraisal. Neurosci. Lett. 496, 147–151. 10.1016/j.neulet.2011.03.066 21457755

[B43] Zebrowitz L. A. Franklin R. G. Jr. Boshyan J. (2015). Face shape and behavior: implications of similarities in infants and adults. Pers. Individ. Differ. 86, 312–317. 10.1016/j.paid.2015.06.036 26217067 PMC4513367

[B44] Zebrowitz L. A. Montepare J. M. (2008). Social psychological face perception: why appearance matters. Soc. Pers. Psychol. Compass 2, 1497–1517. 10.1111/j.1751-9004.2008.00109.x 20107613 PMC2811283

